# Putative parapoxvirus-associated foot disease in the endangered huemul deer (*Hippocamelus bisulcus*) in Bernardo O’Higgins National Park, Chile

**DOI:** 10.1371/journal.pone.0213667

**Published:** 2019-04-17

**Authors:** Alejandro R. Vila, Cristóbal Briceño, Denise McAloose, Tracie A. Seimon, Anibal G. Armién, Elizabeth A. Mauldin, Nicholas A. Be, James B. Thissen, Ana Hinojosa, Manuel Quezada, José Paredes, Iván Avendaño, Alejandra Silva, Marcela M. Uhart

**Affiliations:** 1 Wildlife Conservation Society Chile, Punta Arenas, Chile; 2 ConserLab, Department of Preventive Medicine, Faculty of Animal and Veterinary Sciences, Universidad de Chile, Santiago, Chile; 3 Wildlife Conservation Society, Zoological Health Program, Bronx, NY, United States of America; 4 Ultrastructural Pathology Unit, Veterinary Diagnostic Laboratory, University of Minnesota, St. Paul, MN, United States of America; 5 Department of Pathobiology, University of Pennsylvania School of Veterinary Medicine, Philadelphia, PA, United States of America; 6 Lawrence Livermore National Laboratory, Livermore, CA, United States of America; 7 Departamento de Áreas Silvestres Protegidas, Corporación Nacional Forestal, Chillán, Chile; 8 Departamento de Patología y Medicina Preventiva, Facultad de Ciencias Veterinarias, Universidad de Concepción, Chillán, Chile; 9 Departamento de Áreas Silvestres Protegidas, Corporación Nacional Forestal, Punta Arenas, Chile; 10 One Health Institute, School of Veterinary Medicine, University of California, Davis, CA, United States of America; East Carolina University, Brody Medical School, UNITED STATES

## Abstract

The huemul (*Hippocamelus bisulcus*) is an endangered cervid endemic to southern Argentina and Chile. Here we report foot lesions in 24 huemul from Bernardo O’Higgins National Park, Chile, between 2005 and 2010. Affected deer displayed variably severe clinical signs, including lameness and soft tissue swelling of the limbs proximal to the hoof or in the interdigital space, ulceration of the swollen tissues, and some developed severe proliferative tissue changes that caused various types of abnormal wear, entrapment, and/or displacement of the hooves and/or dewclaws. Animals showed signs of intense pain and reduced mobility followed by loss of body condition and recumbency, which often preceded death. The disease affected both genders and all age categories. Morbidity and mortality reached 80% and 40%, respectively. Diagnostics were restricted to a limited number of cases from which samples were available. Histology revealed severe papillomatous epidermal hyperplasia and superficial dermatitis. Electron microscopy identified viral particles consistent with viruses in the Chordopoxvirinae subfamily. The presence of parapoxvirus DNA was confirmed by a pan-poxvirus PCR assay, showing high identity (98%) with bovine papular stomatitis virus and pseudocowpoxvirus. This is the first report of foot disease in huemul deer in Chile, putatively attributed to poxvirus. Given the high morbidity and mortality observed, this virus might pose a considerable conservation threat to huemul deer in Chilean Patagonia. Moreover, this report highlights a need for improved monitoring of huemul populations and synergistic, rapid response efforts to adequately address disease events that threaten the species.

## Introduction

There is an increasing concern about the potential contribution of diseases in wildlife extinctions, particularly when they interact with other driving factors [1–5]. For example, the effects of infectious pathogens can have devastating effects when population size is small, when multi-host pathogens and reservoir hosts are available, when the infectious agent can survive in an abiotic environment or when disease transmission is influenced by environmental factors or climate change [6–8]. Furthermore, the outcome of an infectious disease depends on intrinsic characteristics of the pathogen that shape morbidity and mortality, ultimately defining severity of illness and the future of affected populations [5, 9, 10].

The huemul deer (*Hippocamelus bisulcus*) is a medium-sized neotropical cervid that is endemic to shrubby habitats and forests in southern Argentina and Chile [11]. Huemul were the most widespread species in Patagonian forests until the 19th century [12], but since that time their range and populations have markedly declined. Contributing factors include habitat loss, poaching, competition with introduced ungulates and susceptibility to livestock diseases [13]. Its current range is now mainly restricted to *Nothophagus* forests in the Andes and periglacial areas surrounding the continental icecaps in Patagonia between 36 and 52° S [14, 15]. At present, huemul are listed as endangered, and fewer than 2,500 individuals remain in fragmented populations in the wild [13, 16]. Studies on huemul health are scarce [17–23]. While most published information is dated and largely anecdotal [24], recent reports suggest disease might be of increasing conservation concern for this species [22, 23].

Bernardo O’Higgins National Park (BONP) in Chile is one of a few remaining strongholds for huemul deer in South America. Some areas of this park are home to the highest densities (4.52 deer/km^2^) of huemul deer across its current range [25]. The remote nature and protection within the park, along with hostile weather and rugged mountainous and coastal topography, are likely significant factors that may help to buffer and protect deer in this area from threats that have led to declines in other regions [26]. Here we describe foot lesions putatively attributed to poxvirus infection and associated morbi-mortality in huemul from BONP.

## Material and methods

### Study area

**Fig 1:**
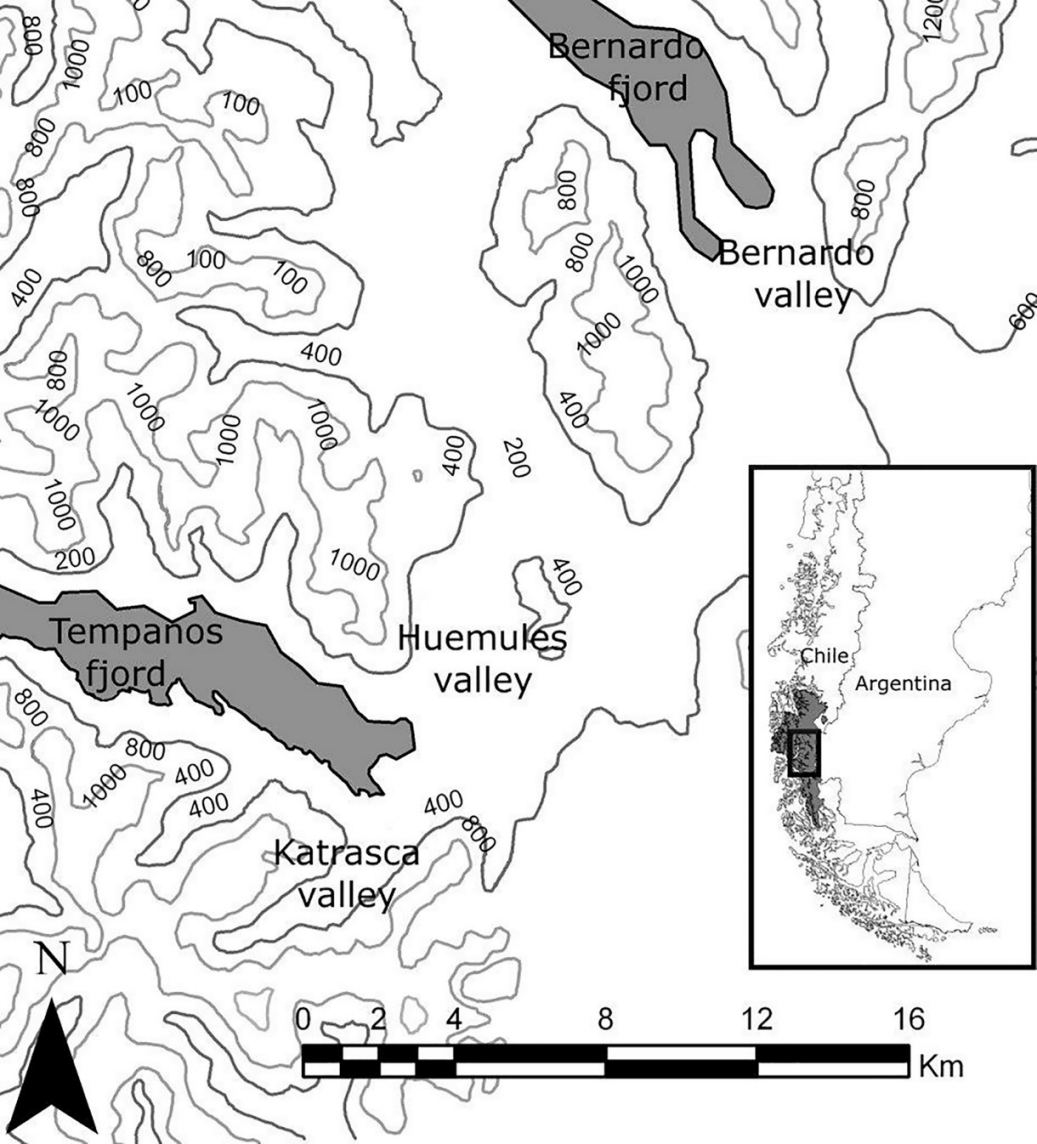
Study area in Bernardo O’Higgins National Park. Locations of the Bernardo, Huemules and Katraska Valleys in the Témpanos and Bernardo Fjords, and elevation contours in m above sea level (a.s.l). The shaded area on the inset indicates the location of the Park, and the rectangle the location of the main map, in Chile.

The study was conducted in public lands at BONP in the Magallanes Region of Chilean Patagonia. This National Park is managed by the Chilean National Forest Service (CONAF). Our study areas were located along the edge of the southern continental icecap in the Huemules (3.2 km^2^), Katraska (5 km^2^) and Bernardo (13.5 km^2^) Valleys (Fig 1). Across this area, the climate is cold and wet. Mean annual precipitation is 4,000 mm and is evenly distributed throughout the year with snowfall from June through August. Annual temperatures average 7–8°C [27]. The vegetation includes periglacial grasslands, grassland–forest ecotones, old-growth forest dominated by *Nothofagus* species and moorlands [26].

Human presence in the area is restricted to Puerto Edén (49°07′34″S, 74°24′48″W), an isolated coastal village with 176 inhabitants located in Wellington Island [28]. In addition, the National Park Service, CONAF maintains a field station in Témpanos Fjord (48°41´33”S; 73°59´21”W). Two park guards have been based at the station throughout the year since 2002.

In 1991, 18 cattle were illegally introduced to Huemules Valley (HV), which affected both huemul abundance and habitat use patterns [26, 29]. By 2001, the cattle population had grown to 31.3 individuals/km^2^ [30], triggering governmental control efforts. While cattle were eliminated from HV by 2004, some animals escaped to neighboring inaccessible areas and continue to be culled opportunistically. Bernardo (BV) and Katraska (KV) Valleys, on the other hand, have always been cattle-free. There are, however, no geographical barriers that prevent animal movements between HV and BV. Following cattle removal, huemul numbers increased in HV [25].

### Field data

Park rangers in Témpanos Fjord have monitored huemul deer and feral cattle presence in HV at least once weekly from 2004. Visits to KV and BV have been less frequent and limited to annual deer abundance surveys conducted once or twice a year. Individual animal identification is made by observation of natural marks, scars, and, for males, antler shape. Observation of huemul foot abnormalities, data and sample collection, and photographs of affected animals reported here were performed in the field by park rangers between 2005 and 2010.

#### Laboratory analysis

External examination, morphometric data recording, photo documentation, gross necropsy examination and tissue sample collection were performed opportunistically on dead huemul by CONAF personnel (Cases #1, #10 and #18). Tissue samples from foot lesions and select internal organs were fixed in 10% neutral buffered formalin prior to routine histologic processing, sectioning at 5 μm, hematoxylin and eosin (HE) staining, and histologic examination. Samples from all cases were examined histologically at Facultad de Ciencias Veterinarias, Universidad de Concepción, Chillán, Chile. Additionally, in 2011, four paraffin blocks from Case #10 (male fawn) containing tissue from affected limbs, were imported to the USA (CITES # 0002245 Chile, #11US033594/9 USA) for additional diagnostics at the Wildlife Conservation Society, Bronx, New York. Following histologic examination, paraffin embedded (FFPE) lesional skin from Case #10 was further analyzed using routine Gram and silver-staining (Warthin-Starry), immunohistochemistry, polymerase chain reaction, microarray testing, and electron microscopy as described below. Laboratory sample disposal protocols at Universidad de Concepción indicate destruction of materials following examination. Therefore, only samples from Case #10 were available for exportation and ancillary diagnostics in the United States.

#### Immunohistochemistry

An immunohistochemical (IHC) assay (DAKO automatic universal staining system) using a rabbit polyclonal antibody against bovine papillomavirus, that is also broadly reactive to canine, feline and equine papillomavirus, and positive and negative controls, was performed on 5 μm sections of formalin-fixed, paraffin-embedded (FFPE) lesional skin (Case #10) (University of Pennsylvania School of Veterinary Medicine, Philadelphia, PA, USA). Briefly, 5 μm, serial sections on negatively charged glass slides were obtained from formalin FFPE tissues. Slides were then heated in a 60°C oven for 1 hour, depararaffinized, then rehydrated with PAR clearant and progressive decreasing grades (from 100% to 95% and to water) of ethanol. Antigen retrieval in citrate buffer (pH ~9.0) and endogenous peroxidases inactivation in hydrogen peroxide (10 min) were performed. Immunolabelling was conducted with a mouse monoclonal, HPV cocktail broad spectrum primary antibody (BioCare, Pacheco, CA, USA), biotinylated goat anti-rabbit and goat anti-mouse secondary antibody, and visualization included incubation with streptavidin conjugated to horseradish peroxidase (15 min) followed incubation with AEC chromogen. Labelled slides were counterstained with hematoxylin (1 min).

#### Transmission electron microscopy

Electron microscopy (EM) was performed on a single, unstained, 5 μm section of FFPE lesional skin (Case #10) mounted on a charged glass slide (Veterinary Diagnostic Laboratory, University of Minnesota). The sample was deparaffinized, rehydrated, and post-fixed in 2.5% glutaraldehyde in 0.1 M sodium cacodylate buffer followed by a second post-fixation in 1% osmium tetroxide in 0.1 M sodium cacodylate buffer (Electron Microscopy Sciences, Hatfield, PA, USA). The sample was dehydrated using a 25%–100% ethyl alcohol gradient. It was then infiltrated and embedded “in situ” with Embed 812 resin (Electron Microscopy Sciences, Hatfield, PA, USA). Embedded tissue was sectioned on a Leica UC6 ultramicrotome (Leica Microsystems, Vienna, Austria). Thin sections (60–70 nm) were obtained and collected onto a 200-mesh nickel grid (Electron Microscopy Sciences, Hatfield, PA, USA). Grids were contrasted with 5% uranyl acetate and Santos’ lead citrate. These preparations were visualized using a JEOL 1400 transmission electron microscope (JEOL LTD, Tokyo, Japan). Images were obtained using an AMT Capture Engine Version 7.00 camera and software (Advanced Microscopy Techniques Corp. Woburn, MA, USA). Image analysis was carried out using ImageJ (NIHR public domain).

#### Polymerase chain reaction (PCR)

DNA was extracted from 50 μm of FFPE sections of lesional skin (Case #10) using the QiAMP DNA FFPE tissue kit, and a protocol adapted for using deparaffinization solution (Qiagen Inc., Valencia, CA, USA). Individual pan-viral polymerase chain reactions (PCR) were performed targeting consensus regions of less than 330 bp for adenoviruses (polymerase gene), herpesviruses (polymerase gene), polyomavirus (VP-1 gene), and flavivirus (NS-5 gene) using previously described methods (Table 1), and appropriate positive and negative controls [31, 32]. Additionally, pan-poxvirus PCR testing (192 bp) was performed on DNA extracted from 5 μm sections (30 μm total) of unstained recut FFPE tissue mounted on charged slides. The primers for this assay were designed to amplify consensus regions using an alignment of the following poxviruses: cowpox, sheeppox, goatpox, deerpox, red deer parapox, bovine popular stomatitis virus, raccoonpox, cetacean poxvirus, dolphin poxvirus, harbor seal parapox, pinniped parapox, Steller sea lion poxvirus, Steller sea lion parapox, sea otter pox, myxomavirus, avipox, canary pox, penguin pox, monkeypox and vaccinia virus as previously described [32]. Amplified products were directly sequenced in the forward and reverse directions (Eton bioscience, Union NJ, USA). All sequences were analyzed, trimmed of their primer sequences, and aligned to generate a consensus sequence that was queried against available sequences in GenBank (National Center for Biotechnology Information, Bethesda, MD, USA).

**Table 1:** Primer sequences and reference methods for PCR assays conducted on samples of deer Case #10.

PCR Assay Target	Amplicon Size (bp)	Primer Name	Primer Sequence (5’to 3’)	Reference and Assay Sensitivity^a^
Adenovirus DNA Polymerase Gene (Nested)	330	•pol-Fouter •pol-Router •pol-Finner •pol-Rinner	•TNMGNGGNGGNMGNTGYTAYCC •GTDGCRAANSHNCCRTABARNGMRTT •GTNTWYGAYATHTGYGGHATGTAYGC •CCANCCBCDRTTRTGNARNGTRA	[33] < 5 copies/reaction
Herpesvirus DNA Polymerase Gene (Nested	250	•DFA •ILK •KG1 •TGV •IYG	•GAYTTYGCNAGYYTNTAYCCR •TCCTGGACAAGCAGCARNYSGCNMTNAA •GTCTTGCTCACCAGNTCNACNCCYTT •TGTAACTCGGTGTAYGGNTTYACNGGNGT •CACAGAGTCCGTRTCNCCRTADAT	[34] < 5 copies/reaction
Polyomavirus VP1 gene	277	•VP1/2F-JO2F •VP1/2R-JO2R	•ATGAAAATGGGGTTGGCCCNCTNTGYAARG •CCCTCATAAACCCGAACYTCYTCHACYTG	[35] sensitivity untested
Poxvirus DNA Polymerase Gene	192	•TSPoxPolF1 •TSPoxPolF2 •TSPoxPolF3 •TSPoxPolF4 •TSPoxPolR1 •TSPoxPolR2	•TATAGAGCGAGTACAGTCATCAAG •GCGAGY ACCTGCA TCAAG •TAYAGAGCTAGTACGTTAATAAAA •TATAGGGCHAGTACKCTTATTAAA •CAIACATTIGGATAYARACTATTATAATC •CGTTIGGGTAYARGCTGTTGTAGTC	[32] 5 copies/reaction
Flavivirus NS-5 gene	270	•Flavi-FWD •Flavi-RVS	•TGYRBTTAYAACATGATGGG •GTGTCCCAICCNGCNGTRTC	[36] 900 copies/reaction

^a^Sensitivity determined using control plasmids

#### Microarray analysis

FFPE tissue sections were removed separately from five glass slides (Case #10) by wetting a scalpel with ethanol and scraping tissue from the surface. Ethanol was removed by centrifugation and samples dried. One ml xylene was added to each sample and vortexed to remove paraffin. Xylene was removed by centrifugation, and remaining tissue was washed in 100% ethanol and dried. Tissue was lysed by incubation with proteinase K at 56°C for one hour, followed by incubation at 90°C to reverse cross-linking. DNA was then extracted from the lysed suspension using the QIAamp cador Pathogen Mini Kit (Qiagen Inc., Valencia, CA, USA) according to the manufacturer’s recommendations. Due to low quantities of total extracted DNA, whole genome amplification was performed using the Repli-g midi kit (Qiagen Inc., Valencia, CA, USA). Samples were amplified at 30°C for 16 hours and purified using QIAquick PCR purification columns (Qiagen Inc., Valencia, CA, USA). Extracted DNA samples were labeled with Cy3 using the NimbleGen One-Color DNA labeling kit (Roche, Inc. Madison, WI, USA). Samples were prepared for hybridization to the Lawrence Livermore Microbial Detection Array (LLMDA v5, 3 arrays x 720K probes) using the NimbleGen hybridization kit LS (Roche, Inc. Madison, WI, USA). This array includes probes designed to detect microbial species with publicly available sequence data obtainable as of December 2011. This layout of the LLMDA platform includes 1,967 bacterial species, 126 archaeal species, 136 fungal species, 94 protozoan species, and 3,111 viruses, and has been previously applied for pathogen surveillance in wildlife [37]. Approximately 15 μg labeled DNA from each sample was added to a separate array, followed by hybridization for 47 hours at 42°C. Arrays were washed using the NimbleGen wash buffer kit and scanned with the MS200 microarray scanner (Roche,Inc. Madison, WI, USA). Data were processed, and potential microbial targets identified using Composite Likelihood Maximization Method (CLiMax) as previously described [38]. Positive probe intensity thresholds were set at the 99^th^ percentile relative to negative control probe intensities and lowered and rerun at the 95^th^ percentile (to broaden possible target pathogen detection). These have been applied as standard thresholds in previous studies using the LLMDA platform for a variety of sample types derived from wild and domestic animals [37, 39] as well as degraded archaeological samples and human lymphoma FFPE tissues [40, 41].

#### Ethics statement

This study was conducted within a cooperation agreement between the Chilean Forestry Agency, Corporación Nacional Forestal–CONAF, and the Wildlife Conservation Society. CONAF is the administrator of terrestrial protected areas in Chile, including Bernardo O’Higgins National Park where the huemul cases occurred. No specific permissions were required for this location or activities which were performed by government personnel following government and forestry agency regulations. As part of their duties, park rangers perform regular observations of huemul deer within the protected area and are authorized to collect samples from dead animals if observed. All animals in this study were dead at the time of necropsy by government personnel. No animals were euthanized in this study.

## Results

Overall, 24 huemul deer with foot lesions were identified between April 2005 and August 2010. Seventy five percent (n = 18) of affected huemul were located in HV; the remainder were found in the more isolated KV (n = 1) and BV (n = 5) (Fig 1). All affected deer displayed similar but varying degrees of clinical signs. These included lameness and soft tissue swelling in one or more limbs just proximal to the hoof or in the interdigital space and, in some cases ulceration of the swollen areas. Some cases spontaneously and completely resolved while others progressed to more severe, proliferative and/or suppurative forms that caused various types of abnormal wear, entrapment, and/or displacement of the hooves and/or dewclaws. These animals showed signs of intense pain such as marked lameness, reluctance to bear any weight on the affected feet, and reduced mobility followed by loss of body condition and prolonged recumbency, which often preceded death.

Deer in HV, but not BV or KV (due to their more rugged landscapes and remoteness), were monitored periodically following the onset of disease. Details on disease evolution and outcome presented in this section are hereafter restricted to the deer in the accessible HV area. Of the 18 affected individuals in HV, four (22.2%) were adult females, ten (55.6%) were adult males, one (5.56%) was a juvenile male, and three (16.7%) were fawns: a female, a male and one of undetermined gender (Table 2). More males (n = 12, 66.7%) than females (n = 5, 27.7%) were found with lesions. Between 25 and 100% of males and 50 to 100% of females in HV were affected, respectively, depending on the year. Furthermore, all juveniles and fawns in HV during the study period were affected. Considering the minimum number of observed deer in HV, morbidity and mortality rates ranged from 40 to 80% and from 0 to 40%, respectively, during the five years of the episode (Table 3).

**Table 2:** Huemul deer with foot lesions in Huemules Valley.

Case #	Date	Sex and Age	Affected limbs		Fate of animal
			Fore	Hind	
**1**	**Apr-05**	**F Ad**	**R**		**Dead**
2	May-05	M Ad			Recovered
3	Sep-05	F Ad			Dead
4	Jun-06	M Ad			Recovered
5	Jun-06	M Juv			Recovered
6	Nov-06	M Ad		R	Unknown
7	Oct-07	M Ad		L	Recovered
8	Oct-07	M Ad		R	Recovered
9	Apr-08	M Ad	L		Unknown
**10**	**Jun-08**	**M Fawn**	**R/L**	**R/L**	**Dead**
11	Aug-08	U Fawn	R	R/L	Recovered
12	Sep-08	F Ad	R/L		Recovered
13	Feb-09	F Ad		L	Unknown
14	May-09	M Ad			Dead
15	Apr-10	M Ad	L		Unknown
16	Apr-10	M Ad	R		Unknown
17	May-10	M Ad		L	Dead
**18**	**Jul-10**	**F Fawn**	**R**	**L**	**Dead**

F = female, M = male, U = unknow, Ad = adult, R = right, and L = left. Cases highlighted in bold have been described extensively in the results section.

**Table 3:** Morbidity and mortality rates attributed to foot disease in huemul deer observed in Huemules Valley (HV).

Year	# of cases	# of deer in the valley	Morbidity (%)	Mortality (%)
2005	3	7	42.9	28.6
2006	3	7	42.9	0.0
2007	2	5	40.0	0.0
2008	4	8	50.0	12.5
2009	2	5	40.0	20.0
2010	4	8	80.0	40.0

Regarding the distribution of lesions, one limb was affected in nine individuals (50%), two limbs in two (11%), and two fawns had either three or four limbs compromised (5.5% in each category). The number of affected limbs was undetermined in the remaining five animals (28%) (Table 2). The outcome of lesions varied. Seven individuals (38.9%) progressed to full recovery, six (33.3%) were found dead within a month of lesion detection (three females and three males), and the outcome was unknown in the remaining five deer (27.8%) because they could not be tracked. Most of the affected individuals in HV were observed during the fall (8 of 16 for which month was recorded), two and five affected deer were observed in the winter and spring, respectively, and only one case was observed in the summer.

Cases were not seen in the remote BV or KV until 2008. Between 2008 and 2010, five adult males (infection rate = 1/18 in 2008 and 4/9 in 2010, all in BV) and a single adult female (1/12 in 2010, only case in KV), were observed with lesions in these valleys. Five of the six deer with foot lesions in KV and BV were recorded in the fall; the remaining one (2008 male) was reported during the spring.

Three huemul carcasses were recovered for examination and sample collection (Cases #1, #10 and #18, respectively; Table 2) by park rangers, and included an adult female (four years old, April 2005) and two fawns (a seven month-old male in June 2008, and an eight month-old female in July 2010) from HV. In all cases where it was possible to recover affected animals or limbs, park rangers described affected tissue as having a strong, fetid smell, suggestive of possible tissue necrosis and/or bacterial or other microbial infection. Additional macroscopic details of the foot lesions presented here are based on observations from available photographs.

### Case #1

**Fig 2:**
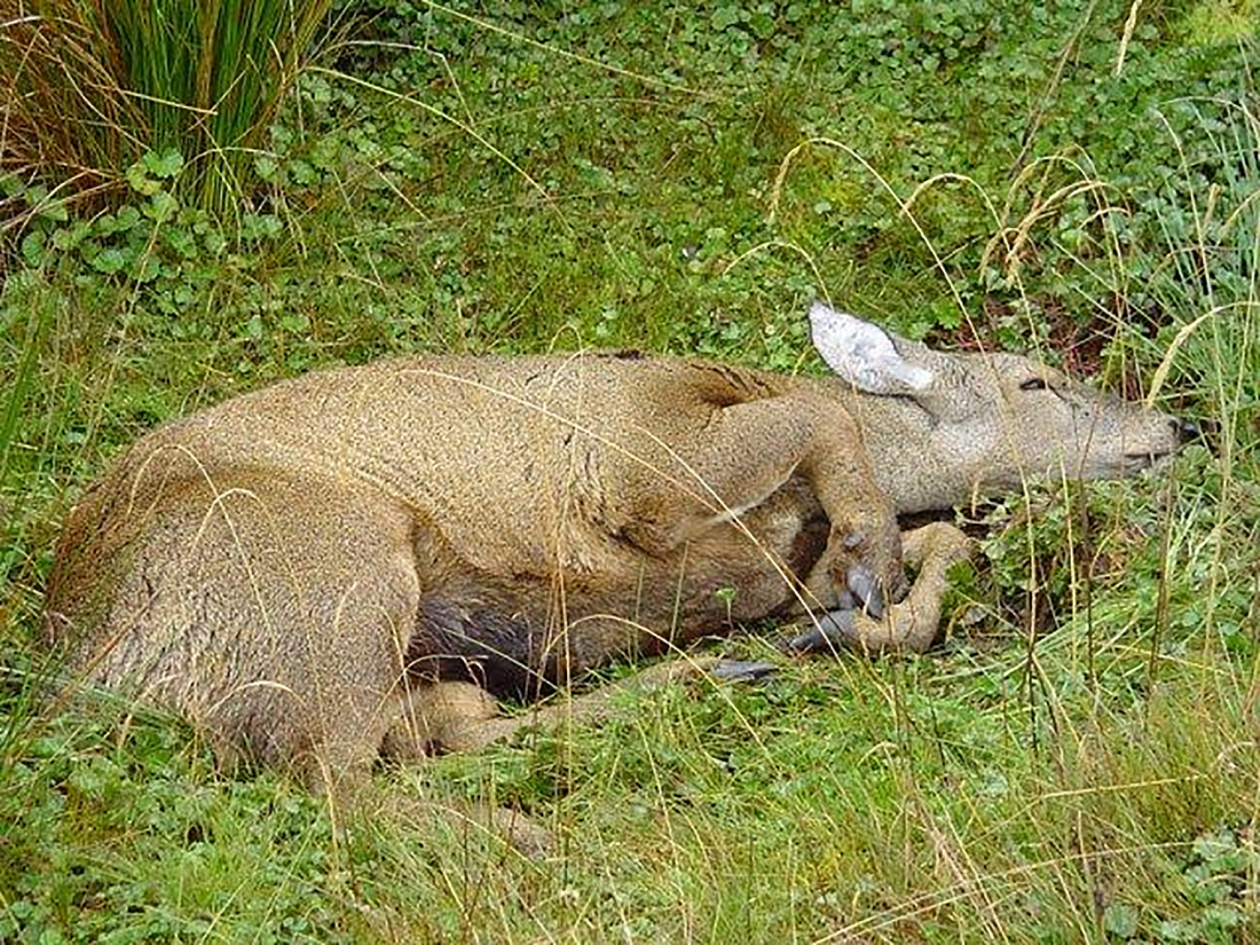
Adult female huemul (Case #1, Table 1), with swelling of right front leg and foot showing signs of pain and recumbency.

**Fig 3:**
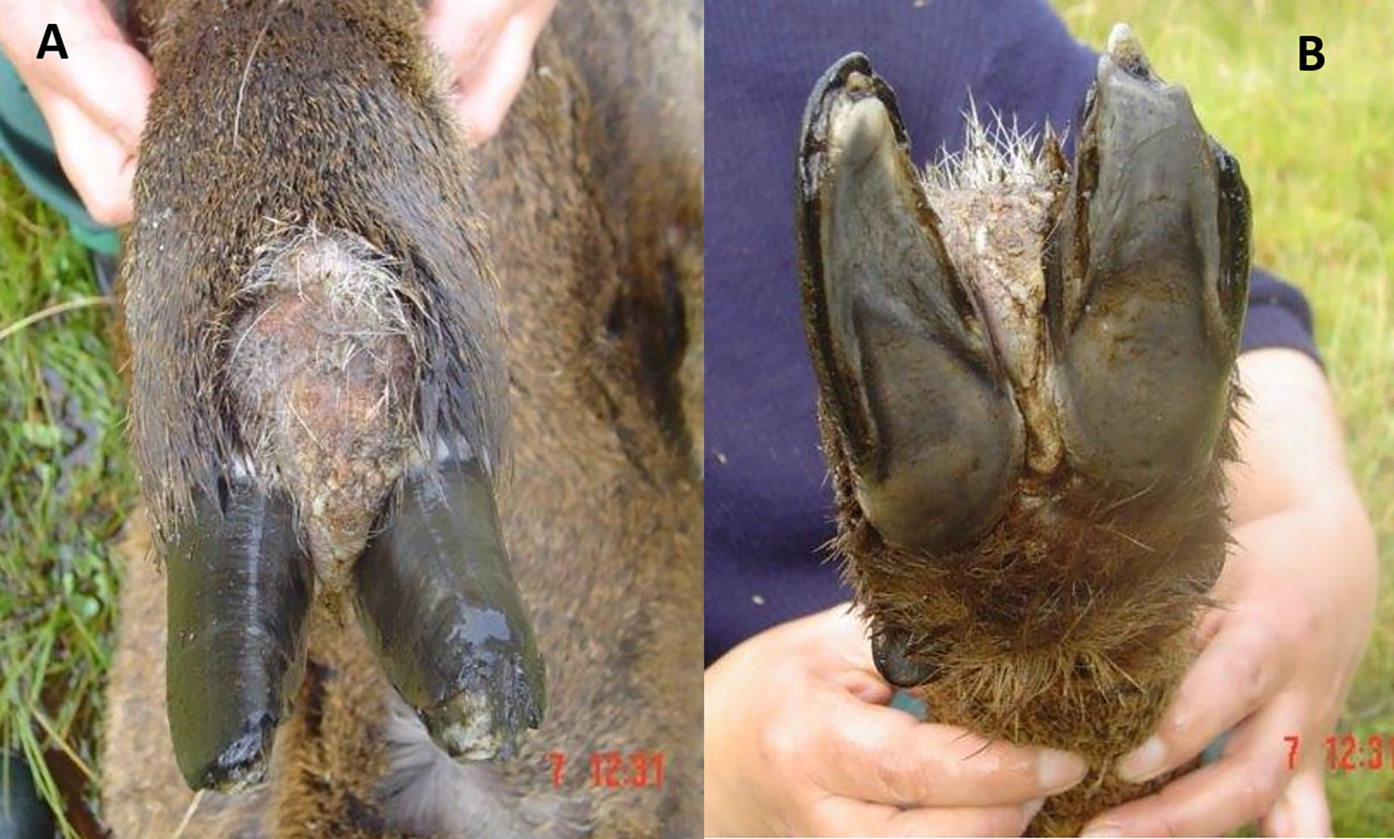
Affected foot (post mortem) of adult female huemul (Case #1, Table 1). A) Dorsal aspect of the right foreleg. A large, roughly round area of soft tissue swelling is present in the interdigital space and immediately proximal and adjacent haired skin, which is focally alopecic. B) Ventral aspect of the right foreleg. The skin is discolored red and gray. Soft tissue swelling has caused moderate separation of the claws, the tips of which are irregularly and abnormally worn.

The first recognized huemul with foot lesions in BONP was an adult female sighted in April 2005 (Case #1, Table 2). The deer was initially observed limping, with marked, diffuse soft tissue swelling of the right forelimb distal to the humero-radial carpal joint, including the carpo-metacarpal joint and foot. Over the next three days the deer was typically recumbent and showed increasing signs of pain (Fig 2). The deer was found drowned in a lagoon four days after initial observations; numerous culpeo fox (*Pseudalopex culpaeus*) prints were seen in the surrounding mud. In addition to generalized swelling of the soft tissues of the foreleg, post mortem findings included a broad based, medially located, regionally extensive area of tissue swelling just proximal to the coronary band that caused partial lateral separation of the claws, and in which the skin was partially alopecic (Fig 3). Additionally, fractures to the tips of the claws on the affected foot, and poor body condition with very small amounts of cavitary and mesenteric adipose tissue were observed.

### Case #10

**Fig 4:**
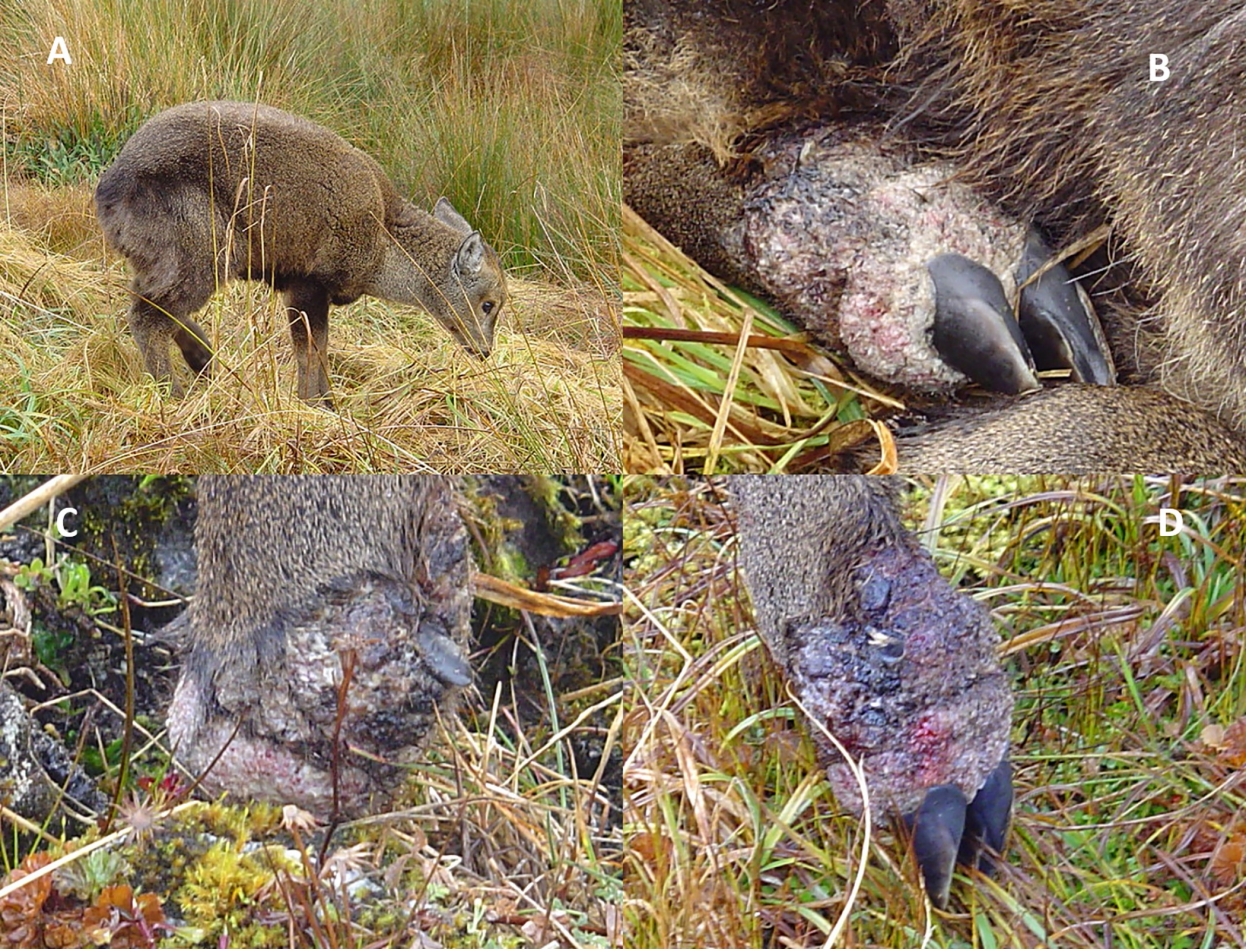
Male huemul fawn case observed in June 2008 (Case #10, Table 1). A) Both left limbs are severely affected. The fawn supported the bulk of its weight on its right limbs, suggestive of pain in the left limbs. B) Close-up of rear left foot. C) Close-up of front foot. D) Close-up of foot and claws.

This was the first case of a young huemul with foot lesions recorded in June 2008 (Case #10, Table 2). This was a male fawn in which all limbs were affected. Lesions consisted of verrucous, proliferative, exophytic tissue that surrounded the base of the hoof, laterally separated the claws, and extended proximally from the level of the coronary band (Fig 4). As in the adult female (Figs 2 and 3), there was a regionally extensive area of alopecia, and there was marked swelling of the soft tissues of the leg and foot. The foot of the left forelimb was more severely affected. Circumferential, proliferative tissue was present around the claws and distal left foreleg and foot (from the claws and coronary band roughly to the mid metacarpal region). Bleeding and drainage of fluid was markedly visible. Lesions on the left rear leg was more restricted to the anterior aspect of the foot; claws on both feet appeared normally worn. The front right limb appeared swollen and draining fluid, with limited proliferative tissue growth. Initially, the fawn displayed signs of severe pain, reduced mobility, and bore most of its weight on its right legs. During the following days, the fawn spent increasing amounts of time recumbent with limited foraging periods. On the fifth successive day of observation, the rear right limb became noticeably swollen. By day ten, the general condition of the deer had declined substantially, and it spent all its time recumbent and was not seen to feed. It was found dead the following day with lesions consistent with fox predation.

**Fig 5:**
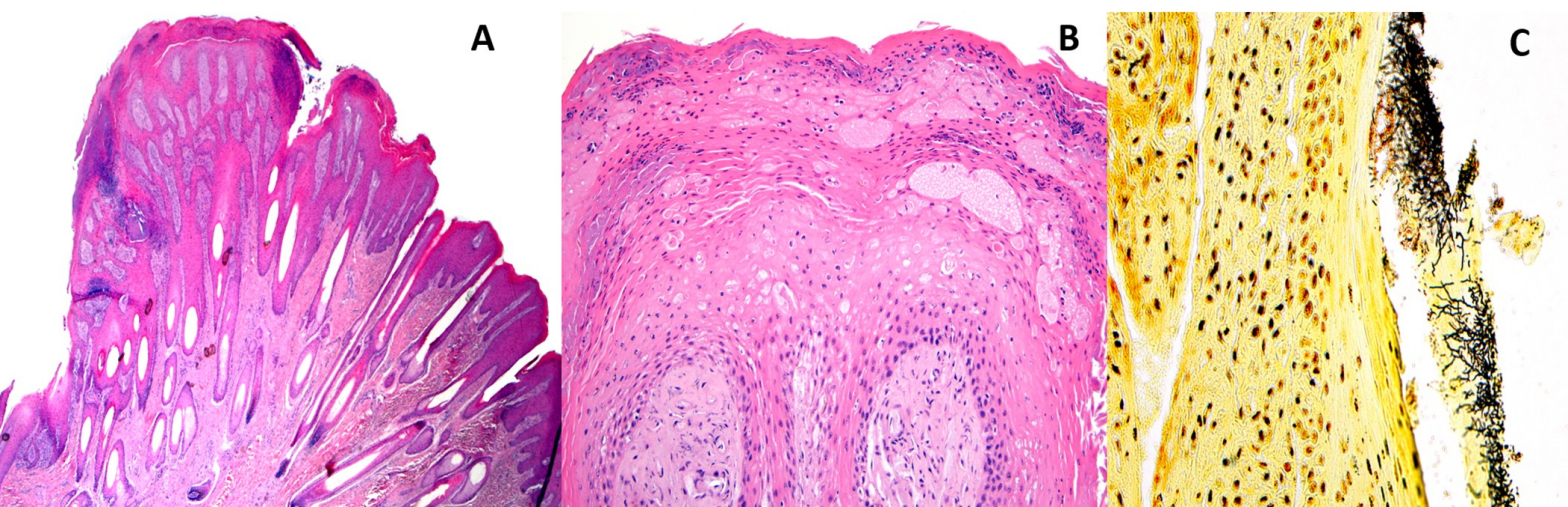
Characteristic histologic changes in affected haired skin from huemul limb (Case #10, Table 1). A) The epidermis is markedly hyperplastic and thrown up into multiple folds. Short rete pegs extend into the subjacent dermis (HE). B) Additional epidermal changes include moderate compact orthokeratotic and parakeratotic hyperkeratosis and mild to severe intracellular edema. Multifocal superficial areas containing smudgy, basophilic material contain degenerate cellular debris admixed with mixed bacteria (HE). C) Myriad long bacteria are stained dark brown and are focally present in this section of an acellular crust overlying the stratum corneum. Short bacilli and cocci that are more lightly stained are present along the base of the crust (Warthin Starry).

Histologic examination of lesions in affected skin from Case #10 (gross lesions seen in Fig 4) consisted of moderate to severe papillomatous to papilliferous epidermal hyperplasia with moderate to severe acanthosis and mild to moderate rete peg formation with maintenance of normal epidermal stratification of the epidermis (Fig 5A). Additional epidermal changes included mild multifocal intercellular edema, mild to moderate ballooning intracellular edema (primarily along the tips of the proliferative epidermis), and moderate compact orthokeratosis and parakeratosis (Fig 5B). Multifocal aggregates of degenerate cells and clusters of mixed bacteria (primarily short coccobacilli) were present along the surface of the thickened, compact stratum corneum. Mixed Gram positive and negative bacteria were highlighted with Gram staining; long, spirochete-like bacteria in the superficial stratum corneum were highlighted with Warthin-Starry staining (Fig 5C). In one area, fungal hyphae (Zygomycete type) were also focally present in the superficial compact stratum corneum. The cellularity of the subepidermal papillary layer of the superficial dermis was mildly increased and contained minimal, multifocal, lymphoplasmacytic inflammation, and the collagen had a slightly ‘smudgy’ appearance multifocally. Hypergranulosis and koilocytes were not seen. Immunohistochemical (IHC) staining for bovine papillomavirus was negative in multiple tissue sections.

**Fig 6:**
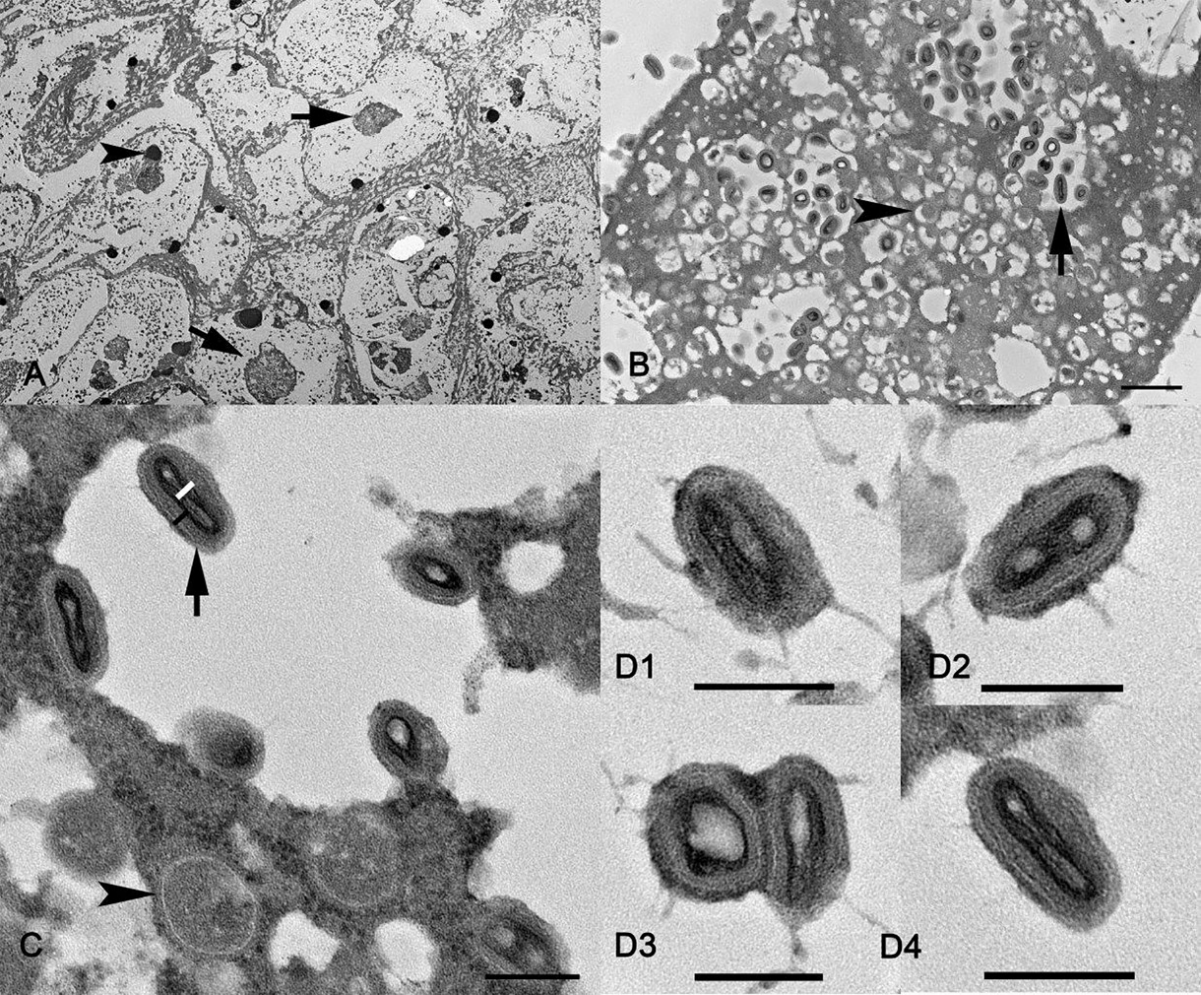
**A-D. Ultrastructure of an affected haired skin from huemul limb (Case #10, Table 1).** Uranyl acetate/lead citrate contrast. A. The cytoplasm of the stratum granulosum cells is clear and distended by myriad poxvirus particles. Within the cytoplasm, some of these cells presented a large electron-dense aggregation consistent with a viral factory (arrows) and fragment of chromatin (arrowhead). **B.** Viral factories were characterized by large aggregation of electron-dense amorphous material admixed with immature (arrowhead), maturing and mature virions (arrow). Bar = 600 nm. **C.** A magnification showed detail of immature virions (arrowhead), which displayed a round shape lined by a radiated membrane. A black bar corresponds to the thickness of the multilayered membrane and a white bar indicates the thickness of the core of a mature virion (arrow). Bar = 200 nm. **D.** Different axis of mature virions. Note that the core is a folded cylinder with horizontal (D1) cross (D2 and D3), and sagittal (D4) sections. Bar = 200 nm.

Electron microscopic examination of skin tissue revealed numerous viral particles in the superficial crust and epidermis. The cytoplasm of these cells was distended and contained a large number of mature viral particles in a clear background (Fig 6A). Within the cytoplasm of epidermal cells were immature, maturing and mature virions admixed in an electron-dense accumulation of amorphous material that correspond to cytoplasmic viral factories (Fig 6A and 6B). Mature viral particles (also known as intracellular mature virus) were characterized by the presence of an electron dense core with a distinct concave shape and lateral body covered by a layered membrane (Fig 6C). Mature virions were approximately 189.3 (SD = 14.1) x 141.6 (SD = 52.3) x 85.3 (SD = 6.8) nm. The core of mature viruses displayed a cylindrical folded morphology. The diameter of the core cylinder was approximately 56.4 (SD = 6.4) nm (Fig 6D). The mature virus membrane and core wall were approximately 29.2 (SD = 4.9) nm thick. The lateral bodies were inconspicuous. Mature virions and a mixed population of bacteria and yeast were present in the superficial crust. Virions presented morphology and dimension consistent with virus of the Chordopoxvirinae subfamily.

PCR was negative for herpesviruses, adenoviruses, polyomaviruses, and flaviviruses, and positive for a parapoxvirus (135 bp). BLASTN analysis showed 98% similarity (3 nucleotide difference) to bovine papular stomatitis virus strain BV-TX09c1 (GenBank accession number KM875472.1) and pseudocowpox virus strain FSS742 (GenBank accession number MH169576.1); the next closest match was 96% homology to another pseudocowpox virus strain VR634 (GenBank No. GQ329670.1).

Microarray analysis did not identify any microbial targets at either the 99th or 95th percentile intensity thresholds, relative to negative control probes, at the default setting of at least 20% of probes detected versus the probes expected to a given target. Hybridization data was further examined for viral identification events at low stringency thresholds (95th percentile, minimum of one detected probe); however, viral detection remained negative under these relaxed parameters.

### Case #18

The third carcass recovered was a female fawn observed in July 2010 with lesions on the left rear limb (Case #18, Table 2). Nine days later, the right forelimb became affected. Eighteen days after the first observation of lesions, purulent material was seen in affected sites, and a noticeable decline in body condition was also apparent. On day 24, the deer became permanently recumbent and displayed evidence of respiratory distress. It died on the 26^th^ day of observation. Histological changes in affected tissue from the foot lesions were similar, but perhaps somewhat more proliferative and papillomatous to those in the male fawn (Case #10).

## Discussion

This is the first report of foot disease, putatively attributed to poxvirus, in huemul deer in Chile. The severity of clinical disease was variable, yet in a third of affected animals (at least six individuals), it resulted in complete incapacitation and death. That a minimum of 18 deer were affected in HV (over five years), with morbidity and mortality rates as high as 80% and 40%, respectively, denotes that foot lesions such as those reported here pose a considerable conservation threat for this species.

Due to limited availability of samples, a thorough investigation was only possible in one individual case (Case #10). Notwithstanding, similarities in the clinical presentation and gross lesions, as well as the progression and outcome of disease, suggest a common etiology and/or pathogenesis. Moreover, the disease behaved as a recurrent outbreak with cases observed predominantly in the fall of each year over a five-year period. Recovery in nearly half of the monitored animals suggests that clinical disease was self-limiting in some individuals. While most affected animals were adults, the increased severity of lesions and higher relative mortality in fawns and juveniles indicates that these age groups were the most likely to succumb to the disease once infected and/or were more likely to die from opportunistic predation.

Numerous foot, interdigital, and hoof diseases have been reported in domestic and wild bovidae, cervidae, and pronghorn antelope [42–52]. Those that more closely resemble the gross appearance of huemul deer cases are footrot or infectious pododermatitis caused by bacteria such as *Dichelobacter nodosus* and *Fusobacterium necrophorum* [51], and the more recently described polymicrobial, multi-treponeme infections associated with digital dermatitis (DD) (‘hairy heel wart’) in sheep and cattle, and severe Treponeme-associated hoof disease (TAHD) in elk (*Cervus canadensis*) [46–48, 49, 52]. Common clinical findings in many of the livestock foot diseases and TAHD of elk that were also seen in huemul are obvious pain and lameness. Grossly, the first affected female huemul (Fig 2, Case #1) presented with interdigital swelling, similar to some of the classic lesions in animals affected by DD and TAHD. However, the evolution and appearance of the lesions in this and other huemul was not typical of footrot necrosis or TAHD. In these diseases, necrosis of the interdigital skin is associated with separation of the horn and undermining of the toe or sole in both and hoof growth abnormalities in the latter. These changes were not seen in the affected huemul. In huemul, rather than necrotizing or ulcerative hoof abnormalities, lesions were primarily proliferative, and in no cases was abnormal hoof growth seen. However, chronic stages of DD can be characterized by thick granulation tissue, something that more closely resembled what was seen in some of the BNOP deer, such as Case #10 fawn (Fig 4). The huemul foot lesions we report also appear to differ from digital dermatitis in that DD most commonly presents as a circumscribed moist ulcerative erosive mass along the coronary band or interdigital space on the plantar aspect of the foot (often a rear foot, though some variation can be seen) [45]. Differences between the huemul cases and TAHD in elk were significant. In elk, a predominant feature is hoof deformation and overgrowth; underlying laminitis is also described. Hoof overgrowth was not seen in affected huemul. However, in some cases, loss of hoof structure was associated with exuberant proliferative granulation tissue. Laminitis was not grossly apparent and was not investigated as a contributing lesion in huemul due to sample availability, but more extensive examination of hooves in future cases would be of value. An additional notable difference between TAHD and huemul lesions is that the latter progressively affected several limbs, including fore and hindlimbs, something not commonly seen in TAHD of elk [49, 50, 52].

DD and TAHD are associated with the presence of multiple treponeme-like bacteria in affected tissues. However, the role of treponemes in hoof syndromes is unclear. Multiple trials have failed to fulfil Koch’s postulates for these bacteria as single causal agents, and current publications refer to a probable polymicrobial etiology, including treponemes, with variations in affected species, geographical location and environmental factors [45, 53]. Notwithstanding, there is agreement that treponemes are present in several chronic ulcerative dermatoses, suggesting common virulence factors that contribute to the development of similar clinical symptoms and lesions [45, 46, 50, 54]. In our study, spirochete-like bacteria were found superficially in the single available tissue sample from Case #10 in Fig 4. Despite several trials, we were unable to further characterize the bacteria through molecular diagnostics due to poor sample quality. However, if these were treponemes, the superficial location differs from what is seen in DD and TAHD in that disease-associated treponemes in the latter are typically invasive and located deep within affected tissue [50]. The more superficial placement in this huemul more likely reflects opportunistic colonization by environmental contaminants rather than primary pathogens. The limitations of our investigation, however, preclude ruling out treponemes or other single or polymicrobial bacterial infections as part of the etiology of this disease.

In addition to abnormal hoof growth, a characteristic feature of TAHD disease in elk is osteomyelitis and interphalangeal osteoarthritis associated with broken and sloughed hooves [49, 50]. There is one published report of osteopathological changes in huemul limbs based on skeletal remains found in Argentina [17]. However, comparison of foot bones from BONP cases with elk was not possible because bone structures were not thoroughly inspected nor preserved in BONP diseased deer, and soft tissues were not available for examination in the skeletal specimens examined by Flueck and Smith-Flueck [17]. In Flueck’s report, examined skeletal remains spanned all age groups, without gender bias, and across a wide temporospatial range. Chronicity of lesions led the authors to favor a nutritional (e.g. selenium deficiency) versus an infectious etiology. Selenium and copper deficiency have been documented in TAHD affected and unaffected elk; however, any nutrition deficiency as a possible contributing factor in lesion development in the huemul of that or this current report, and elk remains to be further investigated. Notwithstanding, the short timeline from disease onset to death in the BONP huemul would not have allowed the development of the chronic, extensive bone remodeling that was seen in the Argentina cases described by Flueck and Smith Flueck [17], and a different underlying disease process is considered more likely.

The papillomatous appearance of huemul foot lesions is also reminiscent of those caused by several viruses including papillomavirus, pox or parapox viruses, and possibly foot and mouth disease. Though viral inclusion bodies were not seen on histological examination, electron microscopy of plastic-embedded skin preparations from the single available affected huemul (Case #10), confirmed the presence of viral particles and viral factories. In the latter, the dimensions and morphology of observed viruses and viral particles was consistent with viruses in the Chordopoxvirinae subfamily. Parapoxviruses can be morphologically distinguished from other Chordopoxvirinae in conventional negative staining electron microscopy by their ovoid appearance and the spiral tubule surrounding the virion’s surface, a distinctive diagnostic property of this genus [55]. Unfortunately, this technique can only be applied to unfixed samples, which were not available. Nevertheless, in the existing samples obtained from FFPE tissue mounted on glass slide from Case #10, intermediate stages of this poxvirus replication and assembly were identified. Stages of replication included membrane crescents surrounding a degenerated electron-dense matrix (most likely viroplasm), spherical immature virus enclosing viroplasm, and/or dense nucleoprotein surrounded by lipid bilayers and numerous mature particles viewed along their long or short axis. This is consistent with the entire spectrum of virus intermediates described in Vero cells infected with other parapoxviruses such as ORF virus and the Chordopoxvirinae prototype vaccinia virus [55]. Moreover, the presence of parapoxvirus DNA in Case #10 samples was confirmed by a pan-poxvirus PCR assay that targets shorter amplicon size ideal for recovery from FFPE tissues [32]. Recognizing that our investigation was limited to one animal due to sample accessibility constraints, testing of additional animals to better understand presence/absence and general relevance of viral infection in huemul foot lesions is warranted. Likewise, efforts to more completely characterize the viral sequence and determine whether infection crossed over from domestic cattle or potentially represents a novel virus is a high priority if additional samples become available.

Parapoxviruses, one of eleven genera within the Chordopoxvirinae subfamily, have been reported in a wide variety of wild and domestic mammals including cervids, bovids, camelids, rodents, and pinnipeds [56–59]. Of four known parapoxvirus species, the prototype is ORF virus, which is endemic in most sheep and goat-raising countries. ORF virus has been reported in reindeer (*Rangifer tarandus*) and muskoxen (*Ovibos moschatus*) in Scandinavia [60–62] as well as in chamois (*Rupicapra rupicapra*) and ibex (*Capra ibex*) in Italy [59], and camelids in Asia, Africa and the Middle East [58]. Another member of this genus, Parapoxvirus of red deer was first described in New Zealand [63] and has recently been found in Germany and Italy [59, 64]. The viral DNA in our study was closely aligned with the two additional species in the parapoxvirus genus, bovine papular stomatitis virus and pseudocowpoxvirus, which are mainly found in cattle.

Parapoxviruses are highly contagious and can be transmitted by direct contact between animals or indirectly by environmental contamination. Moreover, transmission between domestic and wild ungulates has been described, as has zoonotic transmission to humans [57, 65–67]. Proliferative pustular lesions have been attributed to parapoxviruses in several wild species. There are, however, very few reports of lesions on feet or limbs, which are considered atypical presentations in domestic animals [68]. Moreover, rarely do parapoxvirus infections become severe, extensive, and fail to spontaneously regress. The exception is disease caused by ORF virus in sheep and goats [57, 59, 68], semi-domesticated reindeer [60] and camels [58]. In cervids, most reports describe nonparapox or orthopox-like viruses in skin lesions of reindeer, mule deer (*Odocoileus hemionus hemionus*), black-tailed deer (*Odocoileus hemionus colombianus*), pudu (*Pudu puda*), and gazelle (*Gazella subgutturosa*) [69–74].

Viral DNA from Huemul Case #10 showed high identity (98%) with a bovine papular stomatitis virus isolated from cattle in the USA and a pseudocowpoxvirus isolated from human samples in Queensland, Australia. To the best of our knowledge, there are no reports of bovine papular stomatitis virus in deer or other wild ungulates, except for a suspect, non-confirmed case in a captive pudu in Chile [75]. Pseudocowpoxvirus infections have been diagnosed in Finnish reindeer (*Rangifer tarandus tarandus*) [76, 77], dromedary camels (*Camelus dromedarius*) in Sudan [58] and water buffalo (*Bubalus bubalis*) in Brazil [78]. In cattle, both papular stomatitis and pseudocowpox cause lesions on the muzzle, lips, oral mucosa and the teats. The lesions resemble those seen with vesicular stomatitis, bovine viral diarrhea and foot and mouth disease [65, 79]. Infection may also be asymptomatic. Similarly, lesions from pseudocowpoxvirus in reindeer and water buffalo are also restricted to erosions and ulcerations of the oral mucosa and tongue [77, 78]. In camels, the disease is characterized by papules that progress into scabs on the lips, muzzle, nares and eyelids and may extend into gum, palate and tongue [58]. Bovine papular stomatitis occurs worldwide in cattle and is usually of little clinical importance. Herd morbidity may be 100% but mortalities are rare. Infection can occur in animals of all ages, with higher incidence in the young. Immunity is of short duration, and reinfections can occur [79]. Pseudocowpoxvirus infections seem to be recurrent in domesticated species like reindeer and camels [58, 77]. Outbreaks in these species and in water buffalo have been associated with weather, age, husbandry, stress and overlap or contact with livestock, contaminated pastures or fomites [58, 77, 78]. Overall, huemul deer cases seem to differ from those described in cattle and semi-wild ungulates primarily in presentation (more proliferative than vesicular), body location (exclusively in feet), and severity (high morbidity-mortality).

It is possible that parapoxviruses behave differently in huemul deer than in other wild or domestic ungulates. It is also feasible that the etiopathogenesis of foot disease in huemul is similar to DD or TADH in that development may be polymicrobial, requiring two or more etiologic agents (viral and/or bacterial) to progress to severe disease. More research is needed to discern the roles of the pathogens identified in our study, as well as to investigate those that cause foot disease in other domestic and wild ruminants. For example, an investigation by Brandt et al [46] found that while bovine papilloma virus was highly prevalent in cattle with DD, the virus was unlikely to play a role in disease development and maintenance. Conversely, the study showed that co-infecting treponemes were actively involved in disease etiology. Importantly however, many foot diseases of livestock that are prevalent worldwide and cause substantial economic losses remain poorly understood despite considerable efforts to elucidate their etiology.

Our inability to confirm viral EM and PCR findings with microarray analysis in Case #10 was unexpected. This could be related to sample quality and viral particle load. Target sequence fragments corresponding to probes present on the array, which may be distinct from the PCR targeted amplicon, might have been degraded beyond the capacity for hybridization. It is more likely, however, that presence of the pathogen was below the limit of microarray detection, which has generally been observed to be 100–1000 genomic copies [80], whereas limit of PCR detection is expected to be closer to a range of 10–100 copies. In addition, it is possible that, while sample preparation techniques sufficiently preserved particle integrity for visual identification by EM, the fixing and subsequent extraction procedures were not amenable to robust hybridization-based DNA detection.

The apparent concentration of huemul cases in HV, the only valley with a period of cattle presence, poses questions about a potential role for environmental contamination and subsequent disease transmission from feral livestock to huemul. However, cattle presence and disease development in deer did not overlap in time or space. Approximately 35 feral cattle were removed from HV between 2001 and 2004, rendering the valley cattle-free prior to the onset of foot disease in huemul deer in 2005. While specific efforts were not made to document abnormalities in culled cattle, many were slaughtered for human consumption and obvious lesions, such as those seen in huemul, were not observed. Similarly, hunters never reported cattle with mouth, face or foot lesions, nor lameness or recumbent animals. In the KV and BV, where cattle were not introduced and never seen, foot disease was documented in huemul but with lower frequency than in HV. This could reflect a true lower prevalence of disease in these valleys or, because of the low periodicity of visits, a detection bias due to decreased observation efforts in these remote locations. That infection began with huemul in HV and then spread through contact with conspecifics in the other valleys is a possibility, though movements of huemul between HV and the other valleys remain unconfirmed [25]. Notwithstanding, the distance between valleys is well within huemul deer reported movement range, 6.7 [81] to 9.0 km [82], and there are no important geographical barriers between HV and BV. Consequently, huemul movements between these valleys are feasible. Other components such as differences in home range size and patterns of movements between sexes [82], and potentially in habitat use, could also have influenced differential exposure, as male huemul were the more affected sex.

Despite significant challenges related to the remote location of BONP, the extreme environmental conditions at the site, and a restricted number of on-site staff who did as much as could be done with limited equipment, no previous necropsy or sampling training, and limited external support, we were able to identify, and in some cases monitor for the first time, the progression of life-threatening foot lesions in huemul deer in Chilean Patagonia. Even though parapoxvirus findings were limited to materials from one case, the potential implications of a disease caused by a highly-contagious and seemingly aggressive virus in this endangered deer should be readily acknowledged. Moreover, foot lesions reported here and recent *Corynebacterium pseudotuberculosis* infections in huemul at Cerro Castillo, Chile [22] highlight the need for improved capacity to detect, respond and potentially mitigate health risks in all remaining huemul populations. For all these reasons, we strongly recommend strengthening collaborations between government agencies, research facilities and NGOs to enable synergistic efforts and rapid response to future disease events threatening huemul deer.
